# Procyanidin B2 Reduces Vascular Calcification through Inactivation of ERK1/2-RUNX2 Pathway

**DOI:** 10.3390/antiox10060916

**Published:** 2021-06-05

**Authors:** Yingquan Liang, Guilan Chen, Feng Zhang, Xiaoxiao Yang, Yuanli Chen, Yajun Duan, Maoyun Yu, Shuang Zhang, Jihong Han

**Affiliations:** 1 Key Laboratory of Metabolism and Regulation for Major Diseases of Anhui Higher Education Institutes, College of Food and Biological Engineering, Hefei University of Technology, Hefei 230601, China; liangyingquan0564@126.com (Y.L.); 2019171305@mail.hfut.edu.cn (G.C.); 2019111418@mail.hfut.edu.cn (F.Z.); yangxiaoxiao@hftu.edu.cn (X.Y.); chenyuanli@hfut.edu.cn (Y.C.); yduan@hfut.edu.cn (Y.D.); 2020800136@hfut.edu.cn (S.Z.); 2 School of Biological and Pharmaceutical Engineering, West Anhui University, Lu’an 237012, China; yumaoyuntjs@163.com

**Keywords:** vascular calcification, procyanidin B2, ERK1/2, RUNX2, apoE^−/−^ mice

## Abstract

Vascular calcification is strongly associated with atherosclerotic plaque burden and plaque instability. The activation of extracellular signal-regulated kinase 1/2 (ERK1/2) increases runt related transcription factor 2 (RUNX2) expression to promote vascular calcification. Procyanidin B2 (PB2), a potent antioxidant, can inhibit ERK1/2 activation in human aortic smooth muscle cells (HASMCs). However, the effects and involved mechanisms of PB2 on atherosclerotic calcification remain unknown. In current study, we fed apoE-deficient (apoE^−/−^) mice a high-fat diet (HFD) while treating the animals with PB2 for 18 weeks. At the end of the study, we collected blood and aorta samples to determine atherosclerosis and vascular calcification. We found PB2 treatment decreased lesions in en face aorta, thoracic, and abdominal aortas by 21.4, 24.6, and 33.5%, respectively, and reduced sinus lesions in the aortic root by 17.1%. PB2 also increased α-smooth muscle actin expression and collagen content in lesion areas. In the aortic root, PB2 reduced atherosclerotic calcification areas by 75.8%. In vitro, PB2 inhibited inorganic phosphate-induced osteogenesis in HASMCs and aortic rings. Mechanistically, the expression of bone morphogenetic protein 2 and RUNX2 were markedly downregulated by PB2 treatment. Additionally, PB2 inhibited ERK1/2 phosphorylation in the aortic root plaques of apoE^−/−^ mice and calcified HASMCs. Reciprocally, the activation of ERK1/2 phosphorylation by C2-MEK1-mut or epidermal growth factor can partially restore the PB2-inhibited RUNX2 expression or HASMC calcification. In conclusion, our study demonstrates that PB2 inhibits vascular calcification through the inactivation of the ERK1/2-RUNX2 pathway. Our study also suggests that PB2 can be a potential option for vascular calcification treatment.

## 1. Introduction

The vascular calcification is a prevalent pathophenotype in aging, atherosclerosis, hypertension, diabetes, heart valve disease, and chronic kidney disease [1,2,3]. Hydroxyapatite deposition to the blood vessel wall produces ectopic calcification [4]. Vascular calcification can be mainly classified as two types: intimal or atherosclerotic calcification (presents in atherosclerotic plaques) and media calcification (mainly present in diabetic patients) [1,4]. The intimal calcification, especially the spotty-like calcification, is also strongly associated with atherosclerotic plaque burden, instability, and an increased risk of myocardial infarction or stroke [5,6].

The smooth muscle cells (SMCs) are the main cell type in vasculature. Increased extracellular phosphate levels induce SMCs differentiate into osteo/chondroblasts-like cells, which may initiate and promote vascular calcification [4,7]. Several regulators are involved in regulating vascular calcification. For example, runt-related transcription factor 2 (RUNX2) is a crucial osteogenic transcription factor that plays a dominant role in the process of calcification [7]. The activation of RUNX2 accelerates SMC calcification, while the specific deletion of RUNX2 prevents it [8,9]. Bone morphogenetic protein 2 (BMP2) is a critical molecule in the regulation of bone formation and vascular calcification [10]. After binding to its receptor, BMP2 increases RUNX2 expression via Smad1, 5, and 8 phosphorylation [11].

Extracellular signal-regulated kinase 1/2 (ERK1/2) participate in the regulation of various cellular processes including cell adhesion, migration, differentiation, proliferation, and survival [12]. Previous studies have demonstrated that ERK1/2 is also involved in vascular calcification. For instance, C1q/tumor necrosis factor-related protein-3 promotes reactive oxygen species production, which in turn activates the ERK1/2-RUNX2 signaling pathway to enhance the phosphate-induced osteogenic transition of SMCs [13]. We also reported that ERK1/2 inhibition reduces vascular calcification, which is attributed to the ERK1/2 inhibition-reduced Wnt signaling pathway [14].

Procyanidin B2 (PB2), as a member of the flavonoid family, can be widely found in plants and food products, such as grapes, cocoa/chocolate, blueberries, and red wine [15]. PB2 has been demonstrated to exhibit a variety of potent biological and pharmacological activities [16,17,18]. Mechanically, PB2 substantially reduces the activity of ERK1/2, Jun-terminal kinase, and p38 mitogen-activated protein kinase, thereby inactivating cyclooxygenase-2 and matrix metalloproteinase 2 to exhibit its anti-inflammatory function or the anti-migration and invasion of SMCs [18,19].

Though the bioactivities of PB2 have been extensively studied, the effects of PB2 on vascular calcification and the underlying mechanisms remain unknown. Therefore, in this study, we determined whether PB2 could inhibit vascular calcification, and the inhibition was completed through the inactivation of the ERK1/2 signaling pathway.

## 2. Materials and Methods

### 2.1. Reagents

PB2 was purchased from MedChem Express (Monmouth Junction, NJ, USA) and Chengdu Biopurify Phytochemicals Ltd. (Chengdu, China) for cell and animal study, respectively. U0126 was purchased from LC Laboratories (Woburn, MA, USA). Mature pEGFP-C2-mitogen-activated protein kinase 1 (MEK1)-mut (C2-MEK1-mut) was purchased from Addgene (Cambridge, MA, USA). Human epidermal growth factor (EGF) protein was purchased from Sino biological Inc (Beijing, China). Rabbit anti-RUNX2, β-catenin, and BMP2 polyclonal antibodies, as well as the GAPDH monoclonal antibody, were purchased from ABclonal Technology (Cambridge, MA, USA). Rabbit anti-AKT, heat shock protein 90 (HSP90), and α-smooth muscle actin (α-SMA) polyclonal antibodies; mouse anti-phospho-AKT (p-AKT, Ser473) monoclonal antibody; and goat anti-mouse IgG (H+L), FITC conjugate and goat anti-rabbit IgG (H+L), rhodamine conjugate antibodies were purchased from Proteintech Group, Inc. (Rosemont, IL, USA). Rabbit anti-phospho-ERK1/2 (p-ERK1/2, Thr202/Tyr204) polyclonal antibody was purchased from Affinity Biosciences (Cincinnati, OH, USA). Rabbit anti-ERK1/2 polyclonal antibody was purchased from Cell Signaling Technology (Danvers, MA, USA). Goat anti-rabbit and mouse IgG (H+L)-HRP antibodies were purchased from Sungene Biotech (Tianjin, China).

### 2.2. Cell Culture

Human aortic smooth muscle cells (HASMCs) were obtained from ATCC and cultured in a DMEM/F12 (1:1) medium supplemented with 10% FBS, 50 μg/mL of penicillin, and streptomycin.

### 2.3. Determination of Calcification in HASMCs and Aortas

To induce vascular calcification in vitro, HASMCs were cultured in a complete DMEM/F12 (1:1) medium supplemented with 3 mM Na_2_HPO_4_/NaH_2_PO_4_ (1:2) (this medium was named the calcification medium or CM) for 14 days. The medium was changed every 2–3 days [11].

To induce vascular calcification ex vivo, male low-density lipoprotein receptor-deficient (LDLR^−/−^) or C57BL/6J mice (~8 weeks old) fed normal chow were euthanized, and thoracic aortas were obtained. Then the aortic rings were prepared and cultured in CM as described in [11].

### 2.4. In Vivo Study

The protocol for animal studies was approved by the Animal Ethics Committee of Hefei University of Technology and conformed to the Guide for the Care and Use of Laboratory Animals published by NIH. The animal studies were reported in compliance with the ARRIVE guidelines.

All mice were purchased from GemPharmatech Co. Ltd. (Nanjing, China). Male apoE-deficient (apoE^−/−^) mice (~8 weeks old) were randomly divided into 2 groups (10 mice/group) and received the following treatment: the control group (Ctrl) was fed high-fat diet (HFD, containing 21% fat and 0.5% cholesterol) and the PB2 group was fed HFD containing PB2 (80 mg/day/kg bodyweight—the dose was mainly determined by previous studies with a moderate change [20,21,22]) for 18 weeks. At the end of experiment, all mice were euthanized, followed by the collection of blood and aorta samples.

Serum samples were prepared from blood for the determination of levels of total cholesterol (CHO), high-density lipoprotein cholesterol (HDL-C), low-density lipoprotein cholesterol (LDL-C), and triglyceride (TG), as well as the activity of alanine aminotransferase (ALT), aspartate aminotransferase (AST), and alkaline phosphatase (ALP) by the automatic biochemical analyzer (Hitachi 3100, Tokyo, Japan).

### 2.5. Alizarin Red S, Von Kossa, Oil Red O, Sirius Red and Immunofluorescent Staining

Calcification in HASMCs and aortic rings was determined by alizarin red S staining, as described in [23]. To semi-quantify the stained dye of alizarin red S in HASMCs, after staining, cells were added to 10% acetic acid and incubated for 5 min to extract the dye. The absorbance of the extraction solution was determined at the 405 nm wavelength by spectrophotometry [24]. The calcification in aortic rings was also determined by von Kossa staining, as described in [25].

The en face aortic lesions and sinus lesions of the aortic root were determined using oil red O staining [23]. The en face aorta was further divided into three main parts: aortic arch (AA), thoracic aorta (TA), and abdominal aorta (ADA), as described in [26]. The collagen content in lesion areas was detected by the Sirius red staining of aortic root cross sections according to the product instructions. The expression of α-SMA, BMP2, RUNX2, p-ERK1/2, and ERK1/2 in aortic samples was determined by immunofluorescent staining [23].

After staining, all the samples (en face aortas or cross-sections of the aortic root) were observed and the images were obtained with a microscope or fluorescent microscope. The images were then put through semi-quantitative analysis by a technician, who was blinded to the experimental design, using a computer-assisted image analysis protocol (Photoshop CS6, RRID:SCR_014199). The en face lesion areas were expressed as percentages of the whole aorta and the sinus lesion areas in the aortic root as μm^2^/section. The mean fluorescence intensity (MFI) of all immunofluorescent images was calculated as described in [27] using morphometry software (IP Laboratory, Scanalytics, Rockville, MD, USA).

### 2.6. Transfection of HASMCs with C2-MEK1-Mut or pEGFP-C2 Vector

HASMCs at ~80% confluence were transfected with C2-MEK1-mut or the empty vector (pEGFP-C2) using Lipofectamine 2000 Transfection Reagent for 24 h, followed by the process of calcification and the corresponding assays.

### 2.7. Western Blot

After culture or plus treatment for 7 days, total cellular proteins were extracted from HASMCs and used to determine the expression of RUNX2, BMP2, p-ERK1/2, ERK1/2, p-AKT, and AKT by Western blot, as described in [28,29]. Briefly, after treatment and washing with cold PBS, cells were lysed with a lysis buffer containing 0.5 mM phenylmethylsulfonyl fluoride and 1% protease inhibitor cocktail, and then they were incubated for 5 min on ice. Lysate was then centrifuged for 10 min at 12,000 rpm at 4 °C, and the supernatant was saved as whole protein extract. The same amount of total proteins (40–60 µg) from each sample was loaded to and separated on a 10–12% SDS-PAGE and then transferred onto a nitrocellulose filter membrane. The membrane was blocked in PBS containing 5% fat-free milk for about 1 h at room temperature, and then it was incubated with primary antibody overnight at 4 °C. After washing with PBS containing 0.5% Tween-20 (PBST) 3 times, the membrane was incubated with horseradish peroxidase-conjugated secondary antibody for 1 h at room temperature followed by 3 washings with PBST. Then the protein bands were visualized with enhanced chemiluminescence using a chemiluminescence imaging system. After images were obtained, the density of each band was quantified by a technician (blinded to the treatments) with the Image J software (NIH, Bethesda, MD, USA, RRID:SCR_003070). The density of target band was normalized to GAPDH or HSP90 in the corresponding sample, and all values (control and test) were normalized to the mean value of the control group.

### 2.8. Statistical Analysis

All the experiments were repeated at least three times. Data are presented as mean ± SEM. Graph Pad Prism 7.0 was used for statistical analysis. Statistical significance is shown as *p*-value obtained via an unpaired Student’s t test for comparing two experimental groups or a one-way ANOVA test for multiple comparisons of more than two groups. *p* < 0.05 was considered statistically significant.

## 3. Results

### 3.1. Procyanidin B2 Attenuates Atherosclerosis in apoE^−/−^ Mice

**Figure 1:**
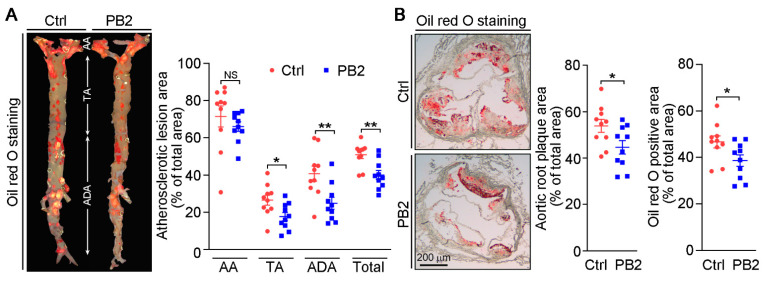
Procyanidin B2 inhibits atherosclerosis in apoE^−/−^ mice. ApoE^−/−^ mice in two groups (10/group) were fed HFD and HFD containing PB2 (80 mg/kg body weight) for 18 weeks. At the end of experiment, aortas and blood samples were collected for the following assays. (**A**) Lesions in en face aortas were determined by oil red O staining, and lesion areas in en face aorta and different segments of aortas were quantified as percentages of the corresponding whole area. AA: aortic arch; TA: thoracic aorta; ADA: abdominal aorta; Total: whole aorta. * *p* < 0.05, ** *p* < 0.01 (10 mice per group were used, and at least three samples from each mouse were taken as technical replicates). (**B**) Aortic root sections were used to determine sinus lesion areas by oil red O staining, and the quantitative results of lesions are presented. * *p* < 0.05 (10 mice per group were used, and at least three samples from each mouse were used as technical replicates).

To detect the effect of PB2 on atherosclerosis and vascular calcification in vivo, apoE^−/−^ mice were randomly divided into two groups and fed HFD (control group) or HFD containing PB2 (PB2 group) for 18 weeks to induce atherosclerosis and calcification [11]. During the treatment, we checked food intake, water consumption, and bodyweight gain weekly, and we found no differences between the two groups. At the end of the experiment, aortas were collected and oil red O staining was conducted for the determination of lesions. Compared to control mice, lesions in en face aorta, thoracic, and abdominal aortas were decreased by 21.4, 24.6, and 33.5%, respectively, by PB2 treatment (Figure 1A). Though lesions in the aortic arch were similar between the control and PB2 groups, PB2 treatment reduced sinus lesions in the aortic root by 17.1% (Figure 1B, middle panel). The reduction of oil red O-positive areas in the aortic root (Figure 1B, right panel) showed that PB2 can inhibit macrophage foam cell accumulation in the arterial wall.

**Figure 2:**
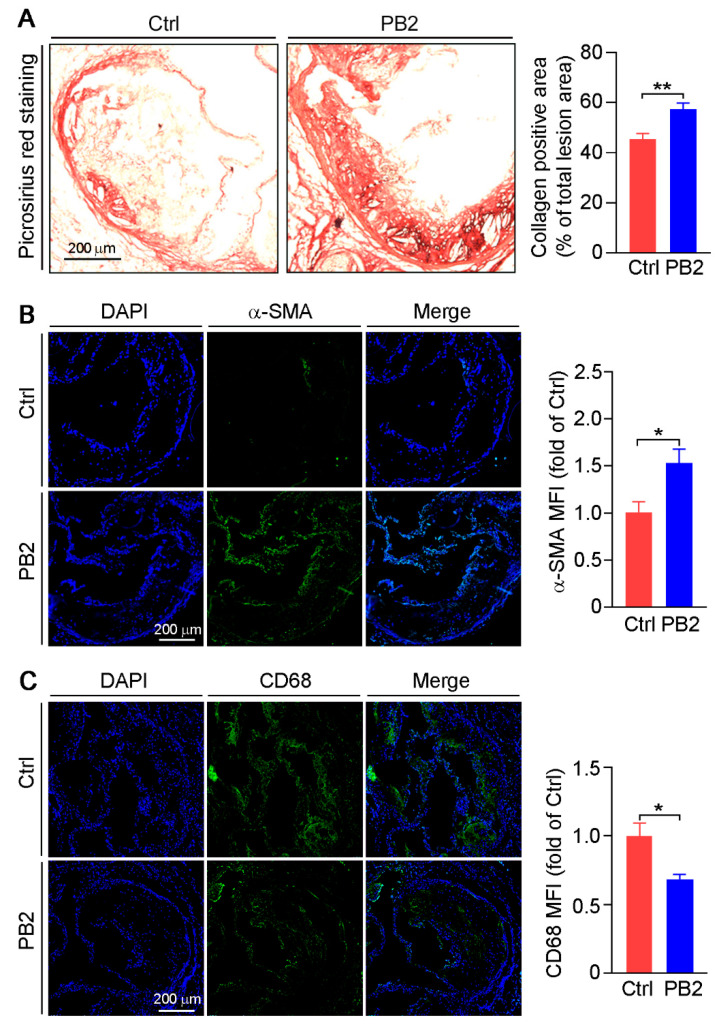
The effects of procyanidin B2 on collagen content and cell composition in lesion areas of aorta root cross sections. (**A**) Collagen content in arterial wall was determined by Picrosirius red staining. ** *p* < 0.01 (10 mice per group were used, and at least three samples from each mouse were taken as technical replicates). The expression of α-SMA (**B**) and CD68 (**C**) was determined by the immunofluorescent staining of cross sections of the aortic root with the quantitative analysis of mean fluorescent intensity (MFI). * *p* < 0.05 (10 mice per group were used, and at least three samples from each mouse were taken as technical replicates).

Lipid homeostasis influences atherosclerosis development. As shown in Table 1, PB2 had little effect on activity of transaminases or ALP, thus indicating no adverse effect of PB2 on the liver. Interestingly, PB2 did not affect lipid profiles (Table 1), suggesting that the reduction of lesions by PB2 is unrelated to its effect on serum lipid levels. Then, we determined the effect of PB2 on collagen content in cross sections of the aortic root using Picrosirius red staining. Compared to the control group, PB2 increased collagen content by 26.4% in lesion areas (Figure 2A). Furthermore, we determined the effect of PB2 on cell composition, primarily SMCs and macrophages, in lesion areas. As shown in Figure 2B, the expression of α-SMA (the marker for contractile phenotype of SMCs) was significantly increased in the PB2 group. Meanwhile, CD68 (the marker for macrophages) expression was substantially decreased (Figure 2C). These results suggest that PB2 reduces atherosclerosis by reducing macrophage accumulation and increasing the amount of SMCs. They also indicate that PB2 may enhance plaque stability by increasing collagen content in the arterial wall.

### 3.2. Procyanidin B2 Reduces Vascular Calcification in apoE^−/−^ Mice

**Figure 3:**
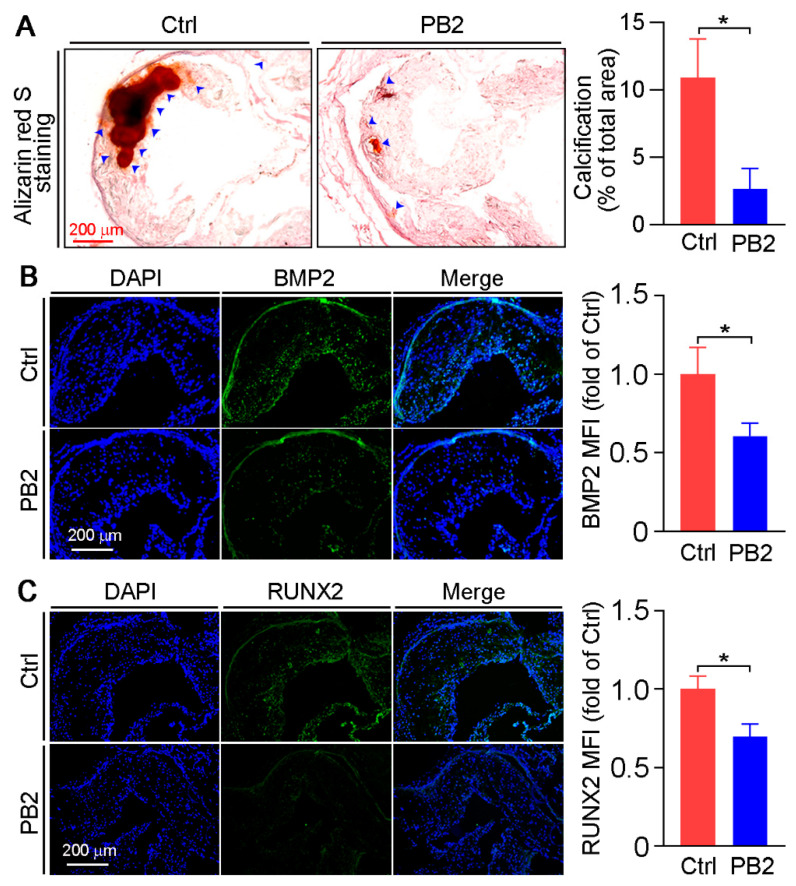
Procyanidin B2 reduces vascular calcification in apoE^−/−^ mice by inhibiting BMP2 and RUNX2 expression. (**A**) Aortic root cross sections were conducted alizarin red S staining to determine calcification with the quantitative analysis of calcification-positive areas. * *p* < 0.05 (10 mice per group were used, and at least three samples from each mouse were taken as technical replicates). The expression of BMP2 (**B**) and RUNX2 (**C**) in aortic root cross sections was determined by immunofluorescent staining with the quantification of positive areas. * *p* < 0.05 (10 mice per group were used, and at least three samples from each mouse were taken as technical replicates).

Calcification is frequently found in advanced atherosclerotic lesions and linked to plaque vulnerability and subsequent acute events. To determine whether the inhibition of atherosclerosis by PB2 is associated with reduced vascular calcification in vivo, we conducted alizarin red S staining with aortic root cross sections. As shown in Figure 3A, severe calcified red regions were determined in the control group. In contrast, the calcified areas in the aorta roots of the PB2 group were substantially decreased by 75.8%.

To further investigate the effect of PB2 on SMC calcification at the molecular level, the expression of BMP2 and RUNX2, the two key regulators for calcification, was detected via the immunofluorescent staining of aortic root cross sections. PB2 significantly inhibited the expression of BMP2 and RUNX2 (Figure 3B,C). Hence, the abovementioned results suggest that PB2 inhibits vascular calcification through the downregulation of BMP2 and RUNX2 expression.

### 3.3. Procyanidin B2 Inhibits Calcification in HASMCs and Aortic Rings

**Figure 4:**
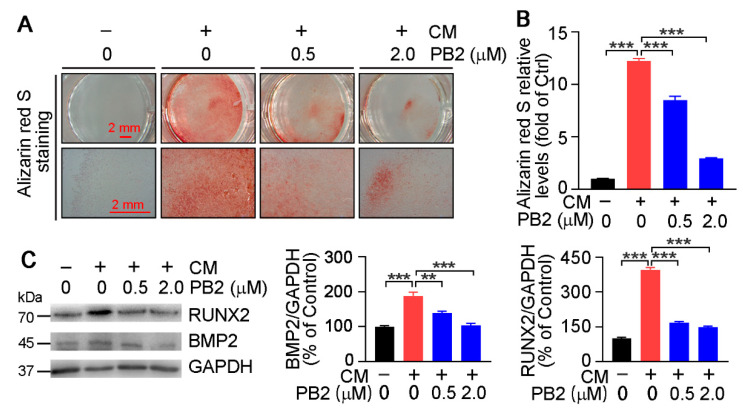
Procyanidin B2 inhibits calcification in vitro. HASMCs were cultured in a normal medium, calcification medium (CM, 3 mM Na_2_HPO_4_/NaH_2_PO_4_ (1:2)), or CM plus PB2 at the indicated concentrations for 2 weeks. (**A**) Calcium deposition was determined by alizarin red S staining, and they images were photographed by the microscope. (**B**) The cells were then added to 10% acetic acid to extract the stained alizarin red S dye. The extraction solution was used to determine absorbance at the 405 nm wavelength with a spectrophotometry. Ctrl, HASMCs were cultured in a normal medium without PB2 treatment. *** *p* < 0.001 (*n* = 3). (**C**) HASMCs were cultured in a normal medium, CM, or CM plus PB2 treatment at the indicated concentrations for 1 week. The protein expression of RUNX2 and BMP2 was determined by Western blot with the quantitative analysis of the band density. ** *p* < 0.01, *** *p* < 0.001 (*n* = 3).

To examine the effect of PB2 on calcification in vitro, HASMCs were cultured in a normal medium, calcification medium (CM, 3 mM Na_2_HPO_4_/NaH_2_PO_4_ (1:2)), or CM plus PB2 for two weeks. The results of alizarin red S staining in Figure 4A show that CM induced severe calcification in HASMCs, but the induction was inhibited by PB2 in a concentration-dependent manner that was confirmed by the semi-quantitative analysis of alizarin red S dye extracted from HASMCs (Figure 4B).

Next, we determined whether PB2 can reduce calcification-induced BMP2 and RUNX2 expression in HASMCs. As shown in Figure 4C, CM induced RUNX2 and BMP2 protein levels, while the induction was substantially reduced by PB2 in a concentration-dependent manner.

**Figure 5:**
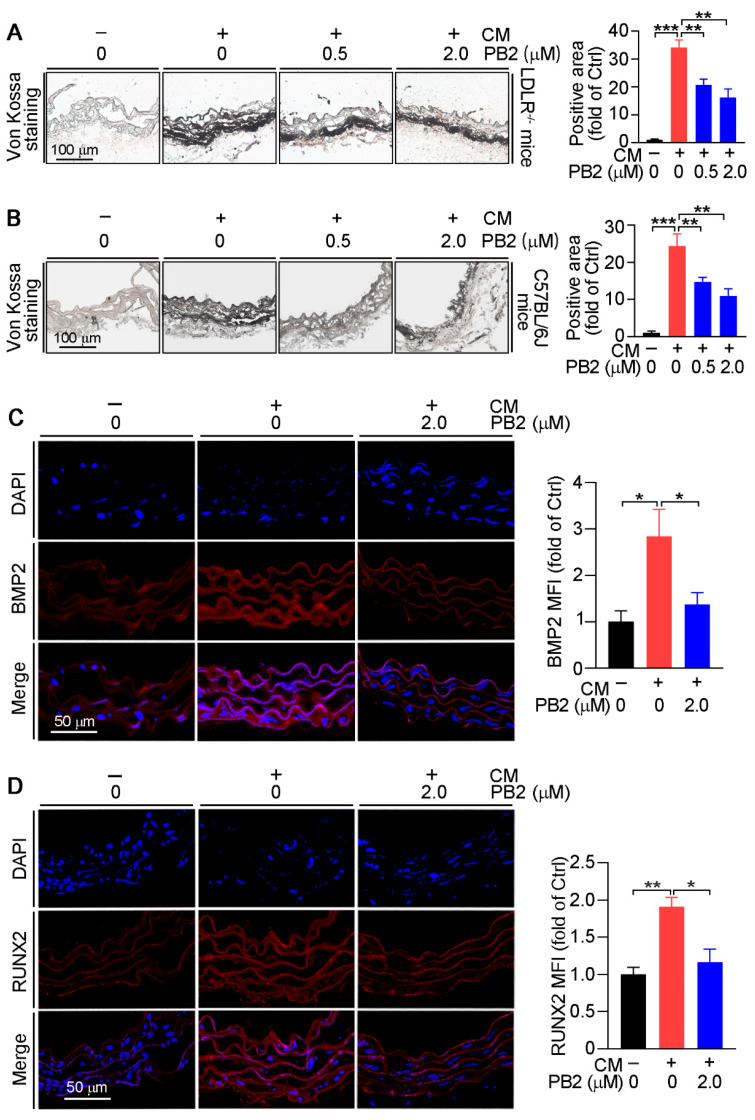
Procyanidin B2 inhibits calcification in the aortic ring. (**A**) Thoracic aortas of LDLR^−/−^ and (**B**) C57BL/6J mice were collected and cut into 0.5-cm rings, followed by culture in a normal medium (Ctrl), CM, or CM plus PB2 at the indicated concentrations for 2 weeks. Calcium deposition in aortic rings was determined by von Kossa staining, and the images were photographed. ** *p* < 0.01, *** *p* < 0.001 (3 mice per group were used, and at least three samples from each mouse were taken as technical replicates). (**C**) BMP2 and (**D**) RUNX2 expression were determined by immunofluorescent staining with the quantification of MFI in the thoracic aortic rings of C57BL/6J mice after cultured in a normal medium (Ctrl), CM, or CM plus PB2 for 2 weeks. * *p* < 0.05, ** *p* < 0.01 (3 mice per group were used, and at least three samples from each mouse were taken as technical replicates).

Furthermore, we confirmed the inhibitory effect of PB2 on calcification ex vivo. The thoracic aortic rings collected from LDLR^−/−^ and C57BL/6J mice were cultured in a normal medium, CM, or CM plus PB2 for two weeks, followed by von Kossa staining. CM also increased the calcification area in aortic rings, whereas PB2 reduced it in a concentration-dependent manner (Figure 5A,B). Meanwhile, CM-activated BMP2 or RUNX2 expression in aortic rings was also blocked by PB2 treatment (Figure 5C,D). Taken together, these results demonstrate that PB2 inhibits calcification in HASMCs and aortic rings with reductions of BMP2 and RUNX2 expression.

### 3.4. Procyanidin B2 Reduces Vascular Calcification by Inactivating ERK1/2 Pathway

**Figure 6:**
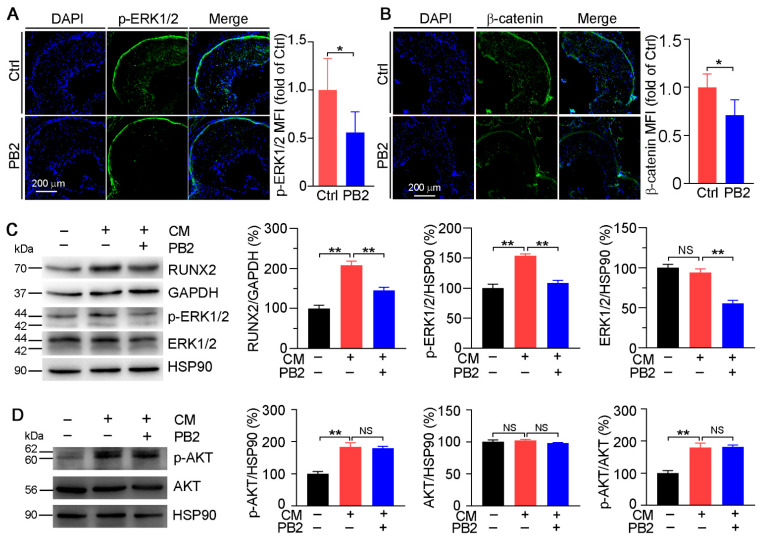
Procyanidin B2 inhibits ERK1/2 activation in vitro and in vivo. The expression of phosphorylated ERK1/2 (p-ERK1/2 (**A**) and β-catenin (**B**) in aortic root cross sections of apoE^−/−^ mice was determined by immunofluorescent staining with the quantification of MFI. * *p* < 0.05 (7 mice per group were used, and at least three samples from each mouse were taken as technical replicates). HASMCs were cultured in a normal medium, CM, or CM plus PB2 (2 µM) for 1 week. The expression of RUNX2, p-ERK1/2, and ERK1/2 (**C**), as well as p-AKT and AKT (**D**), was determined by Western blot with the quantitative analysis of band density or ratio of p-AKT to AKT. ** *p* < 0.01, NS: not significant (*n* = 3).

Previous studies have shown that PB2 can inactivate ERK1/2. Based on the importance of an activated ERK1/2-RUNX2 pathway in SMC osteoporosis differentiation, we anticipated that PB2 may attenuate calcification through the inactivation of the ERK1/2-RUNX2 pathway. Initially, we determined the expression of phosphorylated ERK1/2 (p-ERK1/2, the active form of ERK1/2) in the aorta root cross sections of apoE^−/−^ mice by immunofluorescent staining, and we found p-ERK1/2 was substantially reduced by PB2 treatment (Figure 6A). Previously, we demonstrated that the inhibition of ERK1/2 can reduce vascular calcification by inhibiting β-catenin [12]. Therefore, we tested β-catenin expression in the calcification plaques of apoE^−/−^ mice after PB2 treatment. PB2 was able to dramatically decrease the expression of β-catenin in calcified aorta root sections (Figure 6B), indicating that PB2 may reduce RUNX2 expression via the inhibition of the ERK1/2-Wnt signaling pathway.

In HASMCs, calcification increased p-ERK1/2 but not ERK1/2, indicating the activation of ERK1/2. Meanwhile, RUNX2 expression was promoted in HASMCs in the CM condition. However, PB2 substantially reduced RUNX2, ERK1/2, and p-ERK1/2 expression (Figure 6C).

It has been reported that the AKT signaling pathway can promote SMC calcification by increasing RUNX2 expression [1,30]. In this study, we found that though CM significantly enhanced AKT phosphorylation, PB2 had no effect on it (Figure 6D). Therefore, the inactivation of the ERK1/2 pathway but not the AKT pathway is involved in PB2-inhibted vascular calcification.

### 3.5. Procyanidin B2 Inhibits RUNX2 Expression and Calcification via Inhibiting ERK1/2 Phosphorylation

**Figure 7:**
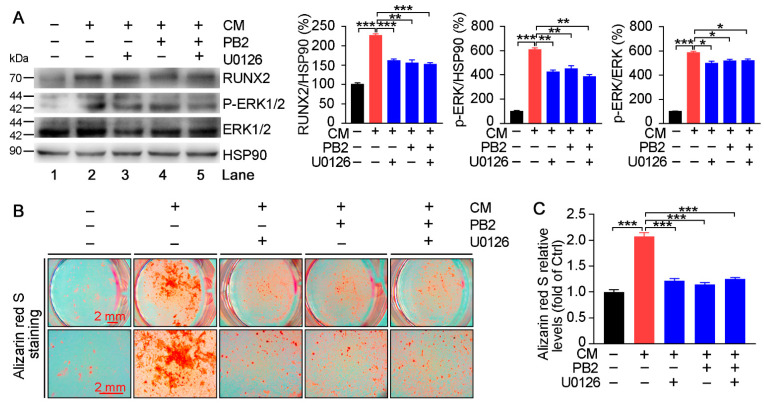
U0126 decreases PB2-inhibited RUNX2 expression and calcification in vitro. HASMCs were cultured in a normal medium, CM, CM plus U0126, or CM plus U0126 and PB2 for 7 (**A**) or 14 days (**B**,**C**). The expression of RUNX2, p-ERK1/2, and ERK1/2 was determined by Western blot (**A**). * *p* < 0.05, ** *p* < 0.01, *** *p* < 0.001 (*n* = 3). Calcium deposition in cells was determined by alizarin red S staining, and images photographed by a microscope (**B**). Cells were then used to extract dye, and the absorbance of extraction solution at the 405 nm wavelength was measured (**C**). *** *p* < 0.001 (*n* = 3).

**Figure 8:**
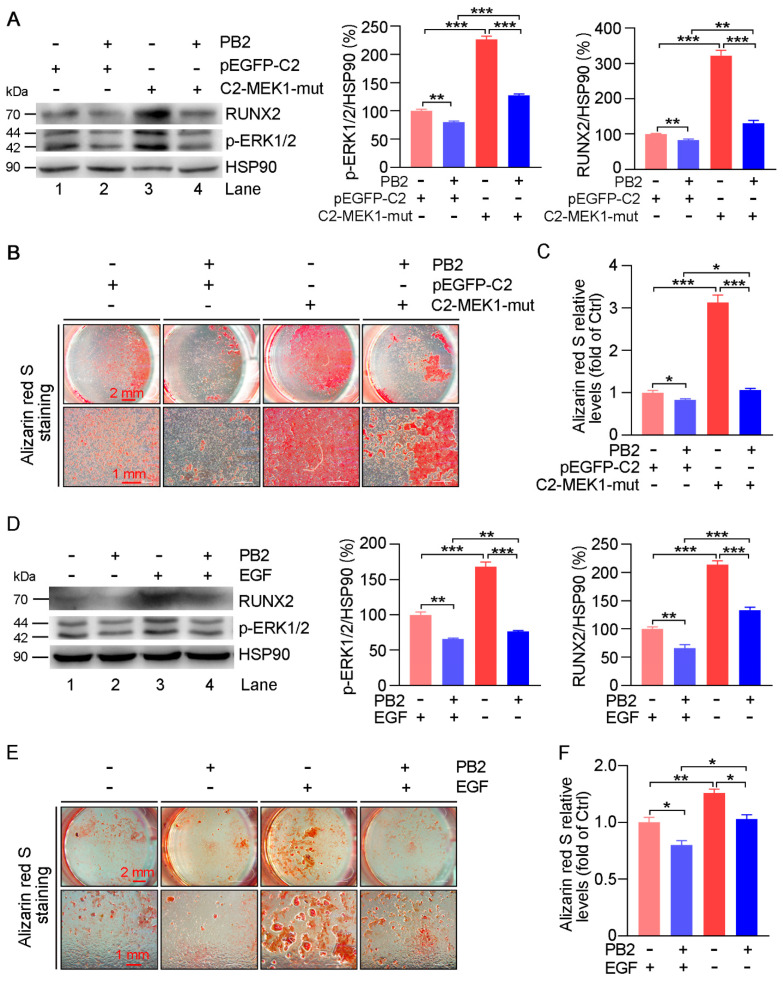
Procyanidin B2 attenuates the activation of ERK1/2 on HASMC calcification. (**A**) HASMCs were transfected with pEGFP-C2 or C2-MEK1-mut for 24 h. The transfected cells had calcification induced in the presence of PB2 (2 μM) for 7 days. The expression of p-ERK1/2, and RUNX2 protein was determined by Western blot. ** *p* < 0.01, *** *p* < 0.001 (*n* = 3). HASMCs were transfected with pEGFP-C2 or C2-MEK1-mut for 24 h and then cultured in a CM medium with PB2 (2 μM) for 2 weeks, followed by alizarin red S staining (**B**) and the quantitative analysis of stained dye (**C**). * *p* < 0.05, *** *p* < 0.001 (*n* = 3). (**D**) HASMC calcification was induced by CM in the presence of PB2 (2 μM) for 2 days, and then the cells were treated with EGF (10 ng/mL) for another 5 days. The expression of p-ERK1/2 and RUNX2 protein was determined by Western blot. ** *p* < 0.01, *** *p* < 0.001 (*n* = 3). HASMCs were cultured in a CM medium containing PB2 (2 μM) for 1 week, and then they received EGF (10 ng/mL) treatment for another week, followed by alizarin red S staining (**E**) and the quantitative analysis of the stained dye (**F**). * *p* < 0.05 (*n* = 3), ** *p* < 0.01.

U0126, an ERK1/2 inhibitor, was used to further confirm the role of the inactivation of ERK1/2 in PB2-inhibited vascular calcification. HASMC calcification was induced by CM condition while receiving PB2 treatment in the presence of U0126. Compared with CM, ERK1/2 expression and phosphorylation and RUNX2 expression were substantially reduced by U0126 treatment (lane 2 vs. 3, Figure 7A). PB2 alone demonstrated similar effects to those of U0126 on the CM-induced expression of RUNX2, p-ERK1/2, and ERK1/2 (lane 4 vs. 2, Figure 7A). Interestingly, no further regulation of these molecules by PB2 was observed in the presence of U0126 (lane 4 vs. 5, Figure 8A). Meanwhile, CM-induced calcium deposition in HASMCs was reduced by PB2 or U0126 alone. However, no further reduction was observed by the co-treatment of PB2 and U0126 (Figure 7B,C). Therefore, the ERK1/2-RUNX2 pathway plays a critical role in PB2-inhibited vascular calcification.

To further explore the role of the inactivation of ERK1/2 on PB2-inhibited vascular calcification, a constitutively active mutant MEK1 expression vector (C2-MEK1-mut) or EGF was used to enhance ERK1/2 phosphorylation. Under the calcification condition, C2-MEK1-mut or EGF markedly increased ERK1/2 phosphorylation and RUNX2 expression (lane 1 vs. 3; Figure 8A,D). PB2-reduced p-ERK1/2 and RUNX2 expression was partially blocked by C2-MEK1-mut or EGF (lane 4 vs. 2; Figure 8A,D). Alizarin red S staining (Figure 8B,E) showed that HASMC calcification was dramatically enhanced by C2-MEK1-mut or EGF, and the induction was decreased by PB2. Similar results were observed with the quantitative analysis of the stained alizarin red S dye (Figure 8C,F). Taken together, the results in Figure 7 and Figure 8 indicate that PB2 can inhibit calcification by inactivating ERK1/2 in HASMCs.

## 4. Discussion

Statins, a group of drugs that help lower serum cholesterol by inhibiting HMG CoA reductase in the liver, are widely used for the clinical treatment of atherosclerosis. However, statins are frequently not available for several reasons, including intolerance, side effects, and, simply, patient preference [31], and they have yet to be shown to effectively inhibit vascular calcification [6]. Recently, nutritional supplements have been recognized as a potential tool to reduce atherosclerosis and calcification [32,33], and nutrients in the diet are much easier to change and follow than other factors.

Procyanidins have attracted considerable attention in the nutrition and pharmaceutical industries due to their health-protective effects observed in vitro and in vivo. Epidemiological evidence indicates that procyanidin consumption can decrease the risk of cardiovascular diseases, diabetes, and cancers [34]. Experimental studies have suggested that procyanidins, extracted from grape seed and litchi pericarp, attenuate atherosclerosis or improve atherosclerotic risk index in vivo [35,36]. The protective effects are generally attributed to the anti-inflammatory and anti-oxidant activities of procyanidins. Especially, procyanidin B2 exerts more biological activity than other B-type procyanidins and epicatechin. For example, PB2 more greatly exerts an anti-oxidative effect than other oligomers, such as procyanidins B1, B4, and B5 [22,37]. Still, the hydroxyl radical and superoxide anion-scavenging activities of PB2 are greater than those of procyanidin B4 and epicatechin extracted from the pericarp tissues of lychee fruit [38]. Due to its anti-oxidative properties, PB2 improves dyslipidemia, hyperglycemia, and oxidative stress in individuals with metabolic syndrome [39]. Mechanically, PB2 inhibits foam cell formation and pyrin domain containing 3 inflammasome activation, and it activates PPARγ to induce M2 phenotype of mouse macrophages. All these beneficial effects may be attributed to the reduction of PB2 on the risk of atherosclerosis [16,40,41,42,43]. However, the anti-atherosclerotic effects of PB2 on apoE^−/−^ mice and the underlying mechanisms remain unknown. In this study, we determined that PB2 not only inhibited the development of atherosclerosis (Figure 1A,B) but also increased collagen or SMC content (Figure 2A,B) and reduced macrophage accumulation (Figure 2C) and calcification (Figure 3A) to enhance the stability of plaques in apoE^−/−^ mice. However, PB2 treatment had no effect on the activity of aminotransferases (Table 1), suggesting its high safety. Meanwhile, PB2 did not affect serum lipid profiles (Table), which indicates that PB2 has limited anti-hypercholesterolemia properties. The reduction of atherosclerosis or vascular calcification by PB2 is completed through mechanisms that are independent of the amelioration of cholesterol/lipid disorders.

Atherosclerotic calcification in an advanced lesion may increase risk of cardiovascular events [44,45]. SMCs play critical roles in mediating intimal calcification by undergoing differentiation to osteoblast-like cells via increasing expression of calcification regulators, RUXN2, and BMP2 [46,47]. RUNX2, a decisive factor and early marker of SMC osteogenic transition and calcification that can be induced by BMP2, increases the expression of multiple osteogenic genes including collagen I, osteopontin, bone sialoprotein, and osteocalcin [11,48]. Here, we demonstrated that PB2-inhibited calcification is associated with reduced BMP2 and RUNX2 expression in vivo (Figure 3B,C) and in vitro (Figure 4C and Figure 5C,D), indicating that PB2 may attenuate calcification by inhibiting BMP2 and RUNX2.

Several pathways involved in SMC transition into osteoblast-like cells have been identified. The ERK1/2 and AKT signaling pathways have been demonstrated to play critical roles in calcification via RUNX2 [7,30,49]. Our previous study also demonstrated that the inhibition of ERK1/2 can activate DKK1 and LRP6 expression, thereby inhibiting the expression of β-catenin to reduce the expression of BMP2 and RUNX2 in vascular calcification [12]. In the present study, we excluded the involvement of AKT in PB2-inhibited calcification because PB2 did not reduce CM-activated AKT (Figure 6D). In contrast, PB2 reduced p-ERK1/2 and β-catenin (Figure 6A,B) in the calcified aortic root of apoE^−/−^ mice. Meanwhile, PB2 reduced p-ERK1/2 expression in phosphate-induced HASMC calcification (Figure 6C).

Both MEK1 and EGF lead to the activation or phosphorylation of ERK1/2 [50,51,52]. We confirmed that constitutively activated MEK1 (C2-MEK1-mut) and EGF can promote p-ERK1/2 and RUNX2 expression and subsequent calcification in HASMCs (Figure 8). The PB2-reduced RUNX2 expression and calcification were partially blocked by C2-MEK1-mut or EGF. Meanwhile, we found that U0126 not only inhibited the expression of ERK1/2 in calcified HASMCs but also had no effects on PB2-reduced RUNX2 expression and calcification (Figure 7), which further confirmed that effects of PB2 on HASMC calcification rely on the inhibition of the ERK1/2-RUNX2 pathway.

In addition to SMCs, macrophages are also believed to be able to promote calcification by stimulating the osteogenic transition of SMCs through cytokine secretion or *trans*differentiating into osteoclast-like cells in the calcifying vessel plaques [53,54]. Recently, studies have reported that transient receptor potential canonical 3 specific deletion in macrophages can reduce calcification and osteogenic features in vivo [55,56]. BMP2 secretion by M1 macrophages maintains constitutive auto/paracrine osteogenic signaling axis [56], while PB2 promotes macrophage M2 polarization and suppresses M1 polarization [42]. In present study, PB2 not only decreased macrophage accumulation but also inhibited BMP2 expression in calcified plaques. We speculate that PB2 may also inhibit the macrophage M1 phenotype to reduce vascular calcification, and further research into the role of PB2 on macrophages in its inhibition of calcification is needed.

## 5. Conclusions

In conclusion, we demonstrated that PB2 can inhibit atherosclerosis, increase plaque stability, and, especially, reduce vascular calcification. The protective effect of PB2 on calcification is associated with the inhibition of the ERK1/2-RUNX2 pathway. These findings suggest that PB2 can work as a potent calcification inhibitor, and have potential clinical implications.

## Figures and Tables

**Table 1:** Effect of PB2 on the activity of aminotransferase and ALP, as well as lipid profiles, in apoE^−/−^ mice.

	Control	PB2
ALT (U/L)	63.36 ± 6.05	60.12 ± 8.56
AST (U/L)	279.00 ± 47.35	270.21 ± 45.53
ALP (U/L)	56.28 ± 4.19	54.96 ± 4.61
TC (mmol/L)	37.32 ± 3.42	41.42 ± 4.12
HDL-C (mmol/L)	0.39 ± 0.03	0.35 ± 0.03
LDL-C (mmol/L)	11.15 ± 1.19	11.22 ± 1.35
TG (mmol/L)	1.16 ± 0.13	1.37 ± 0.17

Data are presented as means ± SEM, *n* = 10 per group. ALT: alanine aminotransferase; AST: aspartate aminotransferase; ALP: alkaline phosphatase; TC: total cholesterol; HDL-C: high-density lipoprotein cholesterol; LDL-C: low-density lipo-protein cholesterol; TG: triglyceride.

## Data Availability

The data presented in this study are available on request from the corresponding author.
